# European LeukemiaNet Response Predicts Disease Progression but Not Thrombosis in Polycythemia Vera

**DOI:** 10.1097/HS9.0000000000000721

**Published:** 2022-05-11

**Authors:** Douglas Tremblay, Andrew Srisuwananukorn, Lukas Ronner, Nikolai Podoltsev, Jason Gotlib, Mark L. Heaney, Andrew Kuykendall, Casey L. O’Connell, Jamile M. Shammo, Angela Fleischman, Ruben Mesa, Abdulraheem Yacoub, Ronald Hoffman, Erin Moshier, Nicole Zubizarreta, John Mascarenhas

**Affiliations:** 1 Tisch Cancer Institute, Icahn School of Medicine at Mount Sinai, New York, USA; 2 Department of Medicine, Hospital of the University of Pennsylvania, Philadelphia, PA, USA; 3 Hematology Section, Department of Medicine, Yale University School of Medicine, New Haven, CT, USA; 4 Stanford University Medical Center, Palo Alto, CA, USA; 5 Herbert Irving Comprehensive Cancer Center, Columbia University Medical Center, New York, USA; 6 Department of Malignant Hematology, H. Lee Moffitt Cancer Center & Research Institute, Tampa, FL, USA; 7 Jane Anne Nohl Division of Hematology, Keck School of Medicine of the University of Southern California, Los Angeles, CA, USA; 8 Division of Hematology/Oncology, Rush University Medical Center, Chicago, IL, USA; 9 University of California, Irvine, CA, USA; 10 UT Health San Antonio Cancer Center, San Antonio, TX, USA; 11 The University of Kansas Cancer Center, Leawood, KS, USA; 12 Department of Population Health Science and Policy, Tisch Cancer Institute, Icahn School of Medicine at Mount Sinai, New York, USA

Polycythemia vera (PV) is a myeloproliferative neoplasm (MPN) characterized by erythrocytosis, constitutional symptoms, and a propensity toward thrombosis and disease progression to postpolycythemia vera myelofibrosis (PPV-MF) or MPN-blast phase (MPN-BP).^1^ Current European LeukemiaNet (ELN) guidelines recommend initiation of cytoreductive therapy in patients >60 years or with a prior thrombosis.^2^ The goal of cytoreductive therapy is to reduce the incidence of thrombosis, although evidence that this intervention accomplishes that goal is largely based on retrospective data.^3^ Common cytoreductive agents used to treat PV include hydroxyurea (HU) and pegylated interferon α-2a (peg-IFNα-2a), in addition to ruxolitinib after HU failure. Novel agents are in development for the treatment of PV, with the ultimate goal of not only reducing thrombotic burden but also preventing disease progression.

To create uniformity in evaluating response to therapeutic intervention, the ELN proposed response criteria in 2009, which were revised in 2013.^4^ These criteria, developed by expert consensus, define a complete response as peripheral blood count normalization for at least 12 weeks in addition to resolution of palpable hepatosplenomegaly, symptom improvement, lack of thrombotic or hemorrhagic events, and bone marrow histological remission (in the case of complete response).^5^ Since their introduction, ELN response criteria have been promulgated as the standard outcome measure for clinical trial evaluating novel therapeutics.^3,6^ However, their utility in predicting relevant PV outcomes in the setting of cytoreductive therapies including peg-IFNα-2a and ruxolitinib, has not been confirmed.

The goal of the present study is to evaluate the prognostic implications of achieving an ELN response in PV patients receiving cytoreductive therapy, as it relates to thrombosis, progression of disease, and death, utilizing a large multi-institutional dataset.

We collected 527 patient records from 10 participating institutions to construct the original database as previously reported.^7^ Institutional review board approval was obtained at all centers before patient data collection. Patients were diagnosed with PV by 2016 World Health Organization criteria and were treated with a cytoreductive agent for at least 12 weeks. Laboratory and spleen measurements were collected from time of cytoreduction until last follow up at approximately 3-month intervals based on availability of data. Attaining a modified ELN response throughout the study period was defined as concurrent white blood cell count (WBC) <10 × 10^9^/L, hematocrit <45%, platelet <400 × 10^9^/L, and lack of palpable splenomegaly.

Outcomes of interest were thrombotic events as recorded in physician notes, and evolution to MF, myelodysplastic syndrome (MDS), and MPN-BP on the basis of a relevant diagnostic bone marrow biopsy and physician documentation within visit notes. Prior thrombosis, cardiovascular (CV) risk factors, and details of cytoreductive agent treatment were documented.

Multivariable Cox proportional hazards methods were used to determine the associations between achieving an ELN response and thrombosis, disease progression, and death. ELN response was analyzed as a time-dependent covariate. All models were adjusted for age, gender, history of prior thrombosis, and the presence of CV risk factors. Median follow-up time was estimated using the reverse Kaplan-Meier method from index time of initiation of cytoreduction. To account for immortal time bias, the landmark analysis approach was used to examine the association between ELN response status and overall survival (OS).

Of the original cohort of 527 patients in the multi-institutional database, 398 were treated with cytoreductive therapy for PV for at least 12 weeks with adequate follow up. Table 1 shows the baseline patient characteristics of the cohort. A total of 119 patients (29.8%) had a prior thrombotic event, which was arterial in 79 patients (19.8%) and venous in 73 patients (18.3%). Thirty-four patients (8.5%) had multiple prior thrombotic events. Aspirin use was documented in 359 patients (90.2%). The majority of patients not receiving aspirin had a prior thrombosis and were presumably receiving anticoagulation.

**Table 1.** Baseline Patient Characteristics

Variable	N = 398
Age, y, median (IQR)	62 (52–69)
Gender, N (%)	
Female	204 (51.3%)
Male	194 (48.7%)
Race, N (%)	
White	314 (78.9%)
Other	33 (8.3%)
Unknown	22 (5.5%)
Asian	15 (3.8%)
Black	12 (3.0%)
Pacific Islander	2 (0.5%)
Months from diagnosis to cytoreduction,median (IQR)	4 (0–25)
Prior thrombosis, N (%)	119 (29.9%)
CVA/TIA	45 (11.3%)
DVT	27 (6.7%)
MI	22 (5.5%)
PE	16 (4.0%)
Hepatic vein thrombosis	14 (3.5%)
Portal vein thrombosis	11 (2.7%)
Other venous thrombosis	11 (2.7%)
Other arterial thrombosis	6 (1.5%)
Splenic infarction	4 (1.0%)
Renal vein thrombosis	3 (0.7%)
Mesenteric vein thrombosis	2 (0.5%)
ELN high risk, N (%)	279 (70.1%)
CV comorbidities, N (%)	252 (63.3%)
Hypertension	167 (42.0%)
Hyperlipidemia	108 (27.1%)
Smoking history	107 (26.8%)
Diabetes	44 (11.1%)
Coronary artery disease	37 (9.3%)
Initial cytoreductive therapy, N (%)	
HU	345 (86.7%)
Pegylated IFNa2a	37 (9.3%)
Ruxolitinib	14 (3.5%)
IFNa2a	2 (0.5%)
Cytoreductive therapy at any time, N (%)	
HU	362 (91.0%)
Ruxolitinib	123 (30.9%)
Pegylated IFNa2a	82 (20.6%)
IFNa2a	5 (1.3%)

CV = cardiovascular; CVA = cerebrovascular accident; DVT = deep vein thrombosis; ELN = European LeukemiaNet; HU = hydroxyurea; IQR = interquartile range; MI = myocardial infarction; PE = pulmonary embolism; TIA = transient ischemic attack.

During a median follow-up of 52.3 months (IQR 27.7–92.0 months), 249 (62.6%) patients attained an ELN response at any time with a median duration of response of 37 weeks (16–74 weeks).

A total of 44 patients experienced a new thrombosis after a median of 3 years (IQR 1.32–5.87), including deep venous thrombosis (DVT) (n = 12), cerebral vascular accident/transient ischemic event (CVA/TIA) (n = 9), myocardial infarction (n = 6), splanchnic vein thrombosis (SVT) (n = 6), pulmonary embolism (n = 5), and other (n = 6). A total of 51 patients experienced disease progression after a median of 7.47 years (IQR 3.83–13.15), including PPV-MF (n = 47), MDS (n = 3), and MPN-BP (n = 1). Thirteen patients died after a median of 6.36 years (IQR 2.67–11.37 years). Cause of death was not captured in this dataset.

In a time-dependent multivariable Cox proportional hazards model, achieving an ELN response was not associated with a reduced hazard of developing a thrombosis (HR, 0.87; 95% CI, 0.41-1.64). Of note, in this model, prior thrombosis was associated with the development of a subsequent thrombosis (HR, 2.67; 95% CI, 1.41-5.06) (Figure 1). Obtaining an ELN response while receiving cytoreductive therapy was significantly associated with a decreased hazard of disease progression (HR, 0.39; 95% CI, 0.17-0.90), although obtaining an ELN response with cytoreductive therapy was not associated with reduced hazard of death (HR, 0.82; 95% CI, 0.23-2.88) (Figure 1). Utilizing a 12-month landmark approach, there was no difference in OS between patients who obtained an ELN response and those who did not (log-rank *P* = 0.11).

**Figure 1.**
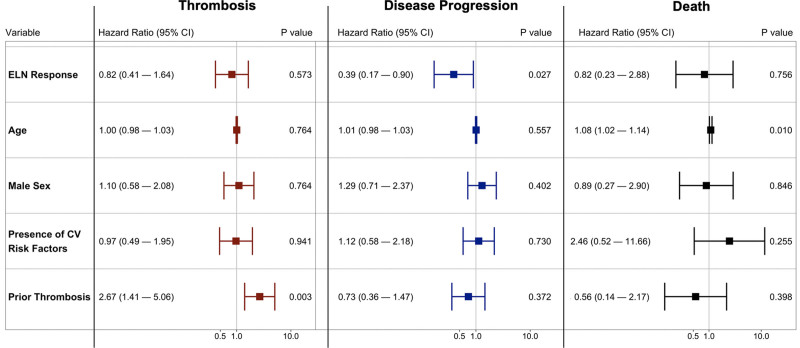
**Independent risk factors for thrombosis, disease progression, and death using a multivariable Cox proportional hazard models with ELN response modeled as a time-dependent covariate.** As shown, obtaining an ELN response was associated with a decrease risk of disease progression, but was not associated with thrombosis or death. CI = confidence interval, CV = cardiovascular, ELN = European LeukemiaNet.

We also examined the contribution of each component of the ELN response to the decreased hazard of disease progression by constructing a time-dependent model that included age, gender, WBC <10 × 109/L, hematocrit <45%, platelet <400 × 10^9^/L, and lack of palpable splenomegaly. WBC response was significantly associated with a decreased hazard of disease progression (HR, 0.26; 95% CI, 0.13-0.52) as was spleen response (HR, 0.27; 95% CI, 0.14-0.56). Hematocrit control was not associated with a change in the hazard of disease progression (HR, 1.80; 95% CI, 0.76-4.27). Interestingly, platelet response was associated with a significantly increased hazard of disease progression (HR, 2.79; 95% CI, 1.31-5.97). Of note, none of the individual components of the ELN response were associated with a decreased hazard of thrombosis.

Previous studies have evaluated the prognostic impact of ELN response criteria on outcomes in both essential thrombocythemia and PV.^8–10^ Our study confirms the results of these by showing that achieving an ELN response is not associated with a decrease hazard of thrombosis. However, in contrast to these previous studies which assessed only HU therapy, we also demonstrate this finding in a population treated with a variety of cytoreductive agents, including peg-IFNα-2a and ruxolitinib. We also show that achieving an ELN response was associated with a decreased hazard of disease progression. However, we acknowledge that it is possible, and in fact likely, that selection bias may explain this finding. In other words, patients who are unable to achieve an ELN response with cytoreductive therapy likely have disease that is at higher risk of progression compared with patients who obtain a response. Therefore, we caution interpreting this finding as justification for increasing the intensity of cytoreductive therapy to achieve an ELN response, as this is not supported by this retrospective study.

Interestingly, when considering the contribution of individual components of ELN response criteria, achieving a platelet response was associated with an increased hazard of progression. Thrombocytopenia is a well characterized feature of MF, where it portends a worse prognosis,^11^ and may be a feature of HU resistance.^12^ Thrombocytopenia is often a harbinger of progressive disease and an eventual diagnosis of MF. Therefore, we hypothesize that the increased frequency of progression associated with platelet response to cytoreductive therapy reflects a PV disease state that is in transition, or already progressed, to PPV-MF.

There are significant limitations in our study that are largely attributable to its retrospective design. Outcomes such as thrombosis and death may be underestimated given reliance on local data entry. Disease progression was clinically defined based on the treating physician and did not require independent hematopathology review. We considered all cytoreductive therapy similarly, but there may be differential effects on thrombosis and progression among HU, peg-IFNα-2a, and ruxolitinib. Sample size limitations and relatively low event rates prevented an analysis to determine if the findings were driven by a specific cytoreductive therapy. Despite these limitations, our retrospective series has several advantages. In particular, the median follow-up of 52.3 months is longer duration than available for many prospective studies.

The results of our study call into question the use of ELN response criteria as an endpoint for novel therapeutics that aim to reduce thrombotic burden, which is the major source of morbidity and mortality in PV patients.^13^ However, ELN response may be an important endpoint in agents that attempt to delay progression of MF. Agents that deplete the PV hematopoietic stem cell pool and possibly affect disease progression, such as the MDM2 inhibitors idasanutlin and navtemadlin, or peg-IFNα-2a, may be more appropriate for trial endpoint evaluation with ELN response criteria.^14,15^ However, for therapies that are designed to reduce thrombotic burden, there remains an unmet need for surrogate endpoints which can accurately predict risk of thrombosis. These findings should be validated, ideally in a prospectively collected dataset. Further work is needed to refine response criteria in order to accelerate and improve the effectiveness of therapeutic development in PV.

## AUTHOR CONTRIBUTIONS

DT, AS, and JM conceptualized the study. DT, LR, NAP, JG, MLH, AK, CO, JMS, AF, RM, AY, RH, and JM contributed patients to the database. DT, AS, NZ, and EM performed the analysis. All authors contributed to article writing and provided final approval of the article.

## DISCLOSURES

DT receives contracted research funding paid to his institution from Astellas Pharma and consulted for and received honoraria from AbbVie and CTI Biopharma. NAP receives contracted research funding paid to his institution from Boehringer Ingelheim, Astellas Pharma, Daiichi Sankyo, Sunesis Pharmaceuticals, Jazz Pharmaceuticals, Pfizer, Astex Pharmaceuticals, CTI biopharma, Celgene, Genentech, AI Therapeutics, Samus Therapeutics, Arog Pharmaceuticals, Kartos Therapeutics and consulted for and received honoraria from Alexion, Pfizer, Agios Pharmaceuticals, Blueprint Medicines, Incyte, Novartis, Celgene, Bristol-Myers Squibb, CTI BioPharma, PharmaEssentia, Constellation pharmaceuticals, Cogent biosciences, AbbVie. JG receives contracted research funding paid to his institution from Deciphera, Novartis, AbbVie, Bristol-Myers Squibb, Kartos, Incyte, Blueprint Medicines and has consulted for and received honoraria from Deciphera, Novartis, Kartos, PharmaEssentia, Cogent Biosciences, Incyte, Blueprint Medicines, and Alkalos. MLH receives contracted research funding paid to his institution from Sierra Oncology, CTI Biopharma, Kartos, Blueprint Medicines, Cogent, and Bristol-Myers Squibb and receives consulted for and received honoraria from CTI Biopharma, Blueprint Medicines, PharmaEssentia and Novartis. AK has receives contracted research funding paid to his institution from Blueprint Medicines, Bristol-Myers Squibb, Prelude, Protagonist and has consulted for and received honoraria from Blueprint Medicines, Bristol-Myers Squibb, CTI Biopharma, Incyte, Novartis, PharmaEssentia, AbbVie, and Protagonist. CLO receives contracted research funding paid to her institution from Genentech, Astex Pharmaceuticals and consulted for and received honoraria from Pfizer, Bristol-Myers Squibb, Astex Pharmaceuticals, and Sionogi. JMS receives contracted research funding paid to her institution from AbbVie, Bristol-Myers Squibb, CTI Biopharma, Kartos, and Incyte and consulted for and received honoraria from Incyte, Bristol-Myers Squibb, and Novartis and serves on the speakers bureau for Incyte and Bristol-Myers Squibb. RM receives contracted research funding paid to his institution from Celgene, Incyte, Abbvie, Samus, Genotech, Promedior, CTI Biopharma, and Constellation and consulted for and received honoraria from Novartis, Sierra Oncology, LaJolla Pharmaceutical and Constellation. AY has consulted for and received honoraria from Incyte, Novartis, Agios, and CTI Biopharma. RH receives contracted research funding paid to his institution from Novartis, Kartos, AbbVie and has consulted for and received honoraria from Protagnoist and serves on Data Safety Monitoring Boards for Novartis and AbbVie. JM receives contracted research funding paid to his institution from Incyte, Janssen, CTI Biopharma, PharmaEssentia, Novartis, Roche, Kartos, Promedior, Merck consulted for and received honoraria from Promedior, Prelude, Galecto, Incyte, Celgene, Kartos, and Geron. All the other authors have no conflicts of interest to disclose.

## SOURCES OF FUNDING

This work was supported by an unrestricted educational grant from PharmaEssentia. PharmaEssentia did not participate in the conception of the project, acquisition of data, analysis of the data, or writing of the article. Research reported in this publication was supported in part by the National Cancer Institute Cancer Center Support Grant P30CA196521-01 awarded to the Tisch Cancer Institute of the Icahn School of Medicine at Mount Sinai and used the Biostatistics Shared Resource Facility. The content is solely the responsibility of the authors and does not necessarily represent the official views of the National Institutes of Health.
